# A six-level clinical autonomy framework for artificial intelligence in dentistry

**DOI:** 10.3389/froh.2026.1836492

**Published:** 2026-06-30

**Authors:** Sohaib Shujaat

**Affiliations:** King Abdullah International Medical Research Center, Department of Maxillofacial Surgery & Diagnostic Sciences, College of Dentistry, King Saud Bin Abdulaziz University for Health Sciences, Ministry of National Guard Health Affairs, Riyadh, Saudi Arabia

**Keywords:** artificial intelligence, autonomous systems, clinical autonomy, decision support systems, dental AI, dentistry

## Abstract

Artificial intelligence in dentistry is rapidly progressing from assistive decision support toward systems capable of executing clinical tasks with increasing autonomy. Despite these advances, the field lacks a structured framework to define, classify, and govern varying levels of clinical autonomy across diagnostic, procedural, and workflow domains in dental practice. This Perspective introduces a dentistry-specific six-level (L0–L5) conceptual clinical autonomy framework characterizing AI systems based on agentic capability, delegated decision authority, human oversight, clinical operating domain, and risk. The proposed taxonomy spans six levels (L0–L5), progressing from human-controlled systems (L0) through assistive (L1), advisory (L2), conditional (L3), and high-autonomy systems (L4), to full operational autonomy within defined clinical contexts (L5). A key inflection point is identified at Level 3, where systems transition from advisory outputs to delegated execution within defined clinical boundaries, marking a shift in responsibility, regulatory classification, and safety requirements. The framework emphasizes functional-level classification, recognizing that autonomy may vary across perception, decision-making, and execution components within hybrid systems. It integrates human-centered considerations, including clinician–AI interaction, transparency, interpretability, and evolving accountability models, while emphasizing inclusive validation and context-aware deployment across diverse patient populations and healthcare settings. By linking autonomy levels to proportional governance and staged translational evaluation, this conceptual framework is intended to support discussion of the safe and responsible integration of AI systems in oral healthcare. The framework has not undergone empirical validation or formal consensus development and should therefore be interpreted as a conceptual taxonomy intended to support future research, regulatory discussion, and refinement.

## 1 Introduction

Dentistry represents a particularly dynamic environment for artificial intelligence (AI) integration. Over the past decade, AI systems have demonstrated high performance in radiographic interpretation, caries detection, periodontal risk stratification, orthodontic treatment planning, prosthetic design, and administrative workflow optimization (1–6). Parallel advances in robotics and digital dentistry, including computer-guided implantology, intraoral scanning, and computer-aided design/computer-aided manufacturing (CAD/CAM) fabrication, have further automated components of clinical practice (1, 7). Yet, despite this technological acceleration, the profession lacks a structured, cross-domain framework to describe the degree of autonomy embedded within these systems (7, 8).

Despite rapid advances in dental AI, much of the published literature remains focused primarily on diagnostic accuracy and task-specific performance evaluation in areas such as radiographic interpretation, caries detection, periodontal assessment, and treatment planning, with comparatively limited attention given to how increasing system autonomy may influence governance, accountability, or clinical oversight structures (2–6). Current robotic applications in dentistry also focus predominantly on implantology and mechanically constrained procedural execution rather than broader cognitive, multimodal, or workflow-integrated autonomy across outpatient dental care settings (7, 8). Moreover, autonomy frameworks derived from automotive automation and surgical robotics were not specifically designed for biologically variable outpatient dental environments characterized by procedural reversibility, heterogeneous workflows, and patient-specific variability (9–13). Consequently, dentistry still lacks a unified framework capable of stratifying varying levels of clinical autonomy across diagnostic, procedural, and workflow domains while linking decisional delegation to proportional oversight and governance.

Autonomy classification has been referenced in the context of robotic dental implant systems, primarily to describe graded levels of mechanical task execution during surgery (8). Such applications, however, remain largely confined to procedural control within intraoperative settings and do not adequately address broader cognitive and workflow-integrated AI functions across dental practice. This absence of a structured autonomy taxonomy creates important gaps in clinical governance, regulatory interpretation, and accountability across AI-enabled dental functions. Without defined levels, it remains unclear when an AI tool should be considered advisory vs. decisional, when regulatory oversight escalates from non-device clinical decision support to software as a medical device, and how liability and ethical accountability shift as machine autonomy increases.

This Perspective proposes a dentistry-specific six-level (L0–L5) clinical autonomy framework for AI systems that adapts existing autonomy taxonomies to the distinctive clinical environment of dentistry. In addition to graded decisional delegation across diagnostic, procedural, and workflow domains, the framework introduces several dentistry-specific considerations, including the Clinical Operating Domain (COD), procedural reversibility, pediatric, geriatric, medically complex, and specialty-specific variability, and functional-level autonomy classification rather than device-level categorization. By aligning levels of autonomy with proportional governance mechanisms, regulatory oversight, and accountability structures, the framework is intended to provide a preliminary conceptual foundation for discussing the safe and responsible integration of AI in oral healthcare.

## 2 Cross-domain foundations of autonomy classification

An autonomy framework for dentistry must build on established models from other safety-critical domains. In the automotive sector, the SAE defines six-levels of driving automation (L0–L5), distinguishing between driver assistance, conditional automation requiring human takeover, and full automation within defined operational limits (9). This taxonomy clarifies role delegation, supervision expectations, and the concept of an Operational Design Domain (ODD).

In healthcare, medical robotics frameworks have proposed six graded levels of autonomy (L0–L5) based on the extent of independent task execution, human supervision, and regulatory accountability. These levels range from teleoperated systems (L0) to fully autonomous robotic systems (L5), explicitly linking increasing decisional delegation to ethical, legal, and regulatory escalation (10). Building on this broader foundation, surgical robotics classifications further differentiate assistive, semi-autonomous, and fully autonomous systems according to procedural workflow execution and intraoperative oversight requirements (11). Human factors research further decomposes automation into functional stages, information acquisition, analysis, decision selection, and action implementation, demonstrating that autonomy may vary across cognitive and operational functions rather than existing as a single global property (12, 13).

Dentistry differs structurally in three respects. First, near-term AI autonomy is likely to emerge in cognitive and administrative domains such as triage, documentation, recall planning, and laboratory coordination before fully autonomous intraoral procedures. Second, dental practice combines medical device risk with outpatient service variability across diverse settings. The automotive concept of an ODD therefore requires reinterpretation as a COD, incorporating setting, modality, patient category, procedural scope, contraindications, and reversibility window. This reinterpretation is essential for translating autonomy concepts from engineering into clinically meaningful boundaries.

In dentistry, COD boundaries may differ substantially across specialties, imaging modalities, and patient populations. For example, AI systems developed for radiographic caries detection on bitewing radiographs may operate under different diagnostic thresholds, lesion prevalence, and validation requirements than systems designed for CBCT-based implant planning or orthodontic workflow management (3, 5, 6). Similarly, pediatric dental settings introduce additional considerations related to mixed dentition, craniofacial growth, behavioral variability, and age-specific disease patterns, which may alter both acceptable autonomy thresholds and oversight requirements compared with adult dental care (14–16). For instance, pediatric AI systems may require additional safeguards due to reduced patient cooperation, higher motion-related imaging variability, evolving occlusion, and the need for caregiver-mediated consent and communication. These factors may limit the safe deployment of higher-autonomy systems in pediatric settings compared with adult care. Similar considerations may also apply to geriatric and medically complex populations, where multimorbidity, polypharmacy, cognitive impairment, altered healing capacity, and increased procedural risk may necessitate lower autonomy thresholds, additional clinician oversight, or narrower validated COD boundaries compared with healthy adult populations.

Unlike automotive ODDs, a dental COD must account for biological variability, tissue response, and procedural reversibility. High-autonomy systems applied to orthodontic aligner staging carry a fundamentally different risk-reversibility profile than similar-autonomy systems applied to surgical bone preparation. Consequently, autonomy classification should be applied at the functional task level, rather than at the device level, because autonomy and its associated risk are expressed through specific clinical actions rather than entire systems. Third, existing taxonomies do not explicitly integrate autonomy classification with proportional regulatory escalation (9–12). In dentistry, increasing decisional delegation must correspond to intensified validation, monitoring, and governance requirements (17). Building on cross-domain taxonomies, the proposed L0–L5 framework adapts autonomy classification to the operational realities of dentistry, including outpatient workflow variability, procedural reversibility, pediatric considerations, and heterogeneous autonomy across perception, planning, and execution functions.

## 3 Dentistry-specific six-level autonomy framework (L0–L5)

**Figure 1:**
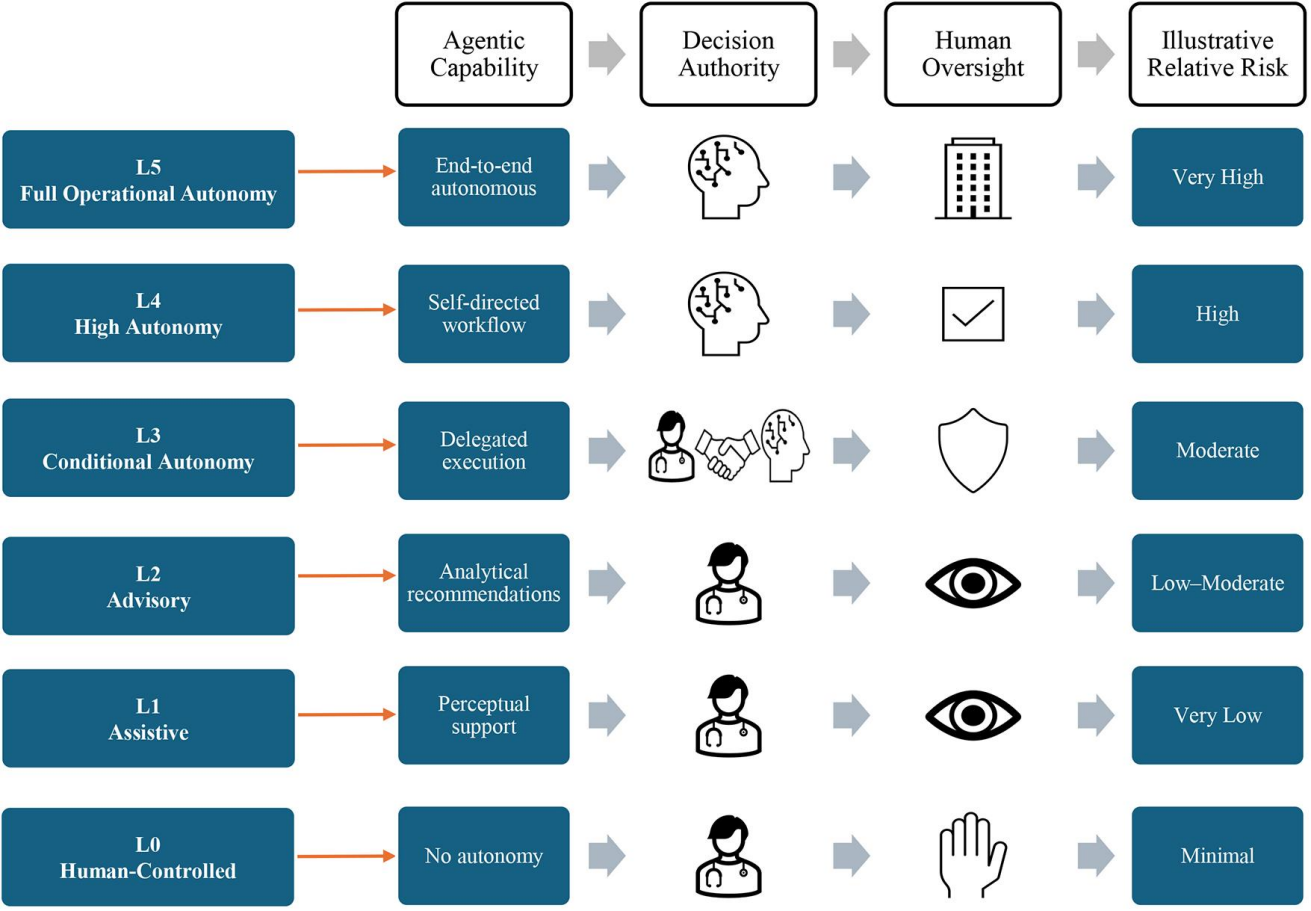
Dentistry-specific six-level (L0–L5) autonomy framework for AI systems in dentistry. The framework presents a conceptual spectrum of increasing delegated operational capability across defined Clinical Operating Domains (CODs), spanning from L0 (Human-Controlled) to L5 (Full Operational Autonomy), based on agentic capability, delegated decision authority, human oversight architecture, and illustrative relative clinical risk. Decision authority progresses from clinician-exclusive control (doctor icon) to shared human–AI authority (handshake) and, within tightly bounded contexts, to AI-dominant operational execution (AI icon). Human oversight evolves from direct control (hand) and continuous monitoring/review (eye) to supervisory override (shield), checkpoint-based review (check mark), and institutional or *post-hoc* governance (building). The dimensions shown represent general progression trends across autonomy levels and are not intended to imply strict linear coupling or that higher autonomy is inherently preferable or clinically superior. Appropriate autonomy depends on procedural risk, reversibility, workflow context, patient factors, and the need for meaningful human control. Hybrid AI systems may contain functional components operating across multiple autonomy levels simultaneously depending on the clinical function being evaluated (e.g., perception, planning, decision-making, or execution).

This section introduces a six-level autonomy framework (L0–L5) for AI systems in dentistry (Figure 1). Importantly, autonomy is classified at the functional task level rather than at the overall device level, recognizing that hybrid dental AI systems may operate at different autonomy levels across perception, planning, and execution functions. The framework classifies systems according to five interrelated dimensions: (a) agentic capability, (b) delegated decision authority, (c) degree of required human oversight, (d) COD boundaries, and (e) clinical risk profile. Together, these dimensions define not only technological sophistication, but also the shifting locus of professional responsibility.

The framework is designed to apply across four major domains of dental practice: diagnosis, treatment planning, procedural execution, and clinical workflow management. Here, the COD defines the validated boundary of operation (setting, patient category, procedure scope, modality availability, contraindications, and reversibility window). Importantly, acceptable autonomy thresholds and oversight requirements may vary across patient populations, including pediatric, geriatric, and medically complex individuals, depending on factors such as procedural reversibility, cooperation, systemic health, cognitive status, and vulnerability to adverse outcomes.

Table 1 summarizes the proposed L0–L5 taxonomy across agentic capability, decision authority, oversight architecture, COD boundaries, and relative clinical risk. Illustrative functional examples are included to demonstrate how varying categories of AI-enabled systems may align with different autonomy levels in practice. These examples may span adjacent autonomy levels depending on implementation context, workflow integration, and degree of delegated execution, and are intended solely for conceptual illustration rather than formal regulatory or performance-based classification.

**Table 1:** Proposed L0–L5 autonomy levels for AI systems in dentistry with illustrative functional examples across diagnostic, planning, procedural, and workflow domains.

Level	Autonomy Label	Agentic Capability	Decision Authority	Oversight Model	Clinical Operating Domain (COD) Scope	Examples (Diagnosis/Planning/Execution/Workflow)	Relative Risk Profile
L0	Human-Controlled (No Autonomy)	No independent reasoning or execution; digital tools function as passive instruments	Clinician retains full authority	Direct, continuous human control	Not applicable; no delegated function	Manual radiograph interpretation; conventional impressions; staff-managed scheduling	Minimal
L1	Assistive (Perceptual or Efficiency Support)	Enhances perception or efficiency (annotations, alerts, stabilization) without independent judgment or multi-step reasoning	Clinician exclusively responsible	Continuous supervision; AI cannot commit actions	Limited to single-task support within defined workflow	AI-assisted radiographic detection and annotation systems providing perceptual or efficiency support without independent clinical judgment; automated charting and documentation tools	Very low
L2	Advisory (Analytical Recommendation)	Analytical recommendation generation, potentially using multimodal inputs, requiring explicit human approval; no independent execution	Clinician approves or rejects AI outputs	Active review before action; no automated system changes	Diagnostic or planning domain within defined clinical constraints	Analytical diagnostic and treatment-planning systems requiring explicit clinician interpretation and approval, including periodontal and radiographic assessment platforms; orthodontic monitoring and cephalometric/treatment-planning systems; AI-assisted CAD/CAM prosthetic design workflows requiring clinician or technician approval; implant-planning and surgical-guide design software requiring clinician approval	Low-Moderate
L3	Conditional Autonomy (Delegated Bounded Execution)	Executes predefined tasks after clinician initiation within a constrained COD; includes mandatory checkpoints before irreversible actions	Shared authority; AI executes under delegated scope	Supervisory monitoring with interruption and override capability	Clearly specified COD (procedure type, patient class, modality availability, reversibility window)	Robotic implant guidance and bounded procedural execution systems operating along clinician-approved pathways with continuous override capability	Moderate
L4	High Autonomy (Domain-Limited Self-Directed Operation)	Proactively plans and executes multi-step workflows within defined COD; monitors outcomes and escalates exceptions	AI primary within bounded domain; clinician supervises outcomes	Checkpoint-based oversight; structured escalation protocols; audit logging required	Narrowly defined COD with validated safety boundaries	Emerging or prototype-level multimodal AI workflow orchestration systems integrating orthodontic monitoring, aligner planning, segmentation, treatment-planning refinement, and downstream manufacturing preparation within predefined clinical boundaries under clinician governance	High
L5	Full Operational Autonomy (Context-Limited)	End-to-end autonomous perception, decision-making, and execution within tightly constrained COD; minimal real-time human involvement	AI assumes operational authority; human governance at institutional level	Post-hoc oversight; external supervision; regulatory governance	Highly restricted COD (e.g., remote screening units; limited-scope care models)	Hypothetical fully autonomous limited-scope dental screening or treatment unit operating within a highly restricted and regulated clinical environment	Very high

***Level 0—Human Controlled Systems:*** Level 0 reflects traditional practice with no AI autonomy. Digital tools function only as passive instruments or storage systems. All perception, interpretation, decisions, and actions remain fully under clinician control. Examples include manual radiograph interpretation, conventional impressions, and staff-managed scheduling. Risk from the machine is negligible because no independent system action occurs. Accountability rests entirely with the clinician.

***Level 1—Assistive AI:*** Level 1 systems enhance perception or efficiency without independent judgment. They may highlight radiographic findings, automate documentation, or provide robotic stabilization, but they do not generate treatment recommendations or execute actions that commit clinical changes. The clinician maintains full authority and continuous oversight. Risk remains low because clinicians remain cognitively engaged throughout the process. Benefits are primarily efficiency and perceptual support.

***Level 2—Advisory AI:*** Level 2 introduces analytical recommendations that require explicit clinician approval. Systems may generate diagnostic probabilities, risk scores, orthodontic adjustments, or prosthetic drafts using multimodal data. Representative applications in oral and maxillofacial radiology include AI-assisted caries detection, periodontal bone loss assessment, cephalometric landmark identification, periapical lesion detection, and CBCT-based anatomical segmentation workflows, where systems provide analytical support while final interpretation remains clinician-controlled. Decision-making authority remains clinician-centered, but cognitive influence increases. Oversight is active rather than manual. Risks include automation bias, though accountability remains predominantly clinician-centered.

***Level 3—Conditional Autonomy:*** Level 3 represents the first point at which AI systems are permitted to act on the clinical environment, rather than merely inform it. Systems at this level execute predefined clinical tasks after clinician initiation, with the dentist supervising and retaining interruption authority. Execution occurs within a clearly specified COD and includes checkpoints before irreversible steps. This level represents the decoupling of clinician intent from clinician action: the clinician authorizes the objective, while the AI performs bounded tool-mediated execution within a validated safety envelope. Representative examples include robotic dental implant systems that provide constrained robotic guidance and bounded procedural execution along clinician-approved trajectories with real-time positional control and automatic safety stops. Additional examples include digitally integrated implant workflows in which, after clinician approval of the surgical plan, the system automatically transfers validated design parameters for surgical guide fabrication within predefined constraints. Authority becomes shared, oversight shifts to supervisory monitoring rather than continuous manual control, and governance requirements increase because execution errors may propagate across interconnected systems. The principal risk is supervisory neglect (automation complacency), where clinicians fail to intervene during out-of-domain or anomalous events despite having override capability.

***Level 4—High Autonomy:*** Level 4 systems can initiate and complete multi-step workflows within a validated COD, escalating exceptions and requiring clinician oversight at defined checkpoints. Examples include integrated multimodal AI workflows combining orthodontic monitoring, aligner planning, segmentation, and manufacturing initiation within predefined clinical boundaries, as well as multimodal planning systems integrating CBCT, intraoral scans, facial scans, and automated segmentation workflows. AI performs highly automated workflow orchestration within validated domain boundaries while clinicians supervise outcomes and exceptions. With increasing autonomy, risk escalates and requires stronger validation, monitoring, and shared accountability across stakeholders.

***Level 5—Full Operational Autonomy in Defined Contexts:*** Level 5 represents end-to-end autonomy within tightly bounded clinical contexts. Systems perceive, decide, and act without real-time supervision. A theoretical example is an autonomous mobile dental unit performing diagnosis and simple treatment in remote settings under remote oversight. Human roles shift toward supervisory and regulatory functions rather than direct procedural involvement. Risk and governance complexity are at their highest. At present, this level remains largely conceptual in dentistry and is included primarily to illustrate potential future trajectories and the importance of defining clear autonomy boundaries.

From a clinical standpoint, this proposed framework may serve as an initial conceptual reference for classifying AI systems according to their level of autonomy and associated risk. It may help clinicians, regulators, and developers discuss oversight expectations, delegated decision authority, and governance considerations across varying autonomy levels. However, the framework has not yet undergone empirical validation or formal consensus development and should therefore not be interpreted as a definitive regulatory or operational classification system.

## 4 Structural features of framework

Across autonomy levels, several consistent patterns emerge. Decision authority progressively shifts from clinician-centered control to AI-centered execution. Human oversight transitions from direct task control to supervisory governance. Data complexity increases from static two-dimensional inputs to multimodal and real-time integration. Risk exposure and regulatory burden rise proportionally with autonomy. Importantly, increasing autonomy should not be interpreted as representing superior clinical design or preferable implementation, as optimal autonomy depends on context-specific clinical, ethical, and safety considerations.

Autonomy may also vary within a single system. Importantly, the five framework dimensions are interrelated but not necessarily linear or uniformly coupled. A system may demonstrate high agentic capability while retaining low delegated authority if clinician approval remains mandatory before execution. Similarly, clinical risk may not always scale proportionally with autonomy level, as risk is also influenced by procedural invasiveness, reversibility, patient factors, and COD constraints. Clinical risk is therefore treated as a related but partially independent dimension influenced by procedural invasiveness, reversibility, patient vulnerability, and workflow context. Accordingly, autonomy classification within the framework is determined primarily by the degree of delegated execution authority and operational independence rather than by any single dimension alone. Figure 1 should therefore be interpreted as a conceptual representation of general escalation trends rather than as a strictly deterministic hierarchy across all dimensions.

Hybrid architectures can operate at different levels across perception, planning, and execution stages. For instance, a diagnostic component may function at Level 2 while a procedural execution module operates at Level 3. For example, an integrated implant-planning workflow may combine multiple autonomy levels within a single clinical system. An AI-assisted radiographic segmentation module that proposes anatomical boundaries while requiring clinician confirmation would function at Level 2 (Advisory AI). Following clinician approval of implant positioning and surgical parameters, a robotic guidance module capable of constrained trajectory execution with real-time override capability would operate at Level 3 (Conditional Autonomy). In this example, the COD would include predefined implant sites, validated imaging modalities, patient eligibility criteria, and interruption safeguards before irreversible drilling steps. Governance requirements would therefore differ across components, with analytical validation emphasized for the Level 2 module and procedural safety, interruption reliability, supervisory oversight, and auditability emphasized for the Level 3 execution component. This example illustrates the importance of functional-level rather than device-level autonomy classification in hybrid dental AI systems.

This highlights the importance of functional classification rather than labeling an entire product with a single autonomy level. To reduce interpretive variability, autonomy assignment should specify function domain, COD boundaries, delegated decision authority, oversight architecture, tool access, fallback behavior, auditability, update policy, and the degree of independent execution permitted within the validated clinical workflow. By formalizing these distinctions, the L0–L5 framework establishes a shared vocabulary for researchers, clinicians, regulators, and industry stakeholders evaluating AI systems in dentistry.

## 5 Preliminary operational rubric for autonomy assignment

To improve reproducibility and reduce interpretive variability, autonomy assignment should follow a structured functional-level assessment process rather than relying solely on narrative interpretation. Classification should be performed separately for each functional component of a system (e.g., perception, planning, execution, and workflow management). In general, systems should be classified according to the highest level of delegated execution achieved within their validated COD. Where dimensions span multiple levels, classification should default to the highest level of delegated execution authority permitted within the validated COD. Five core dimensions should be evaluated during classification: (1) whether the system can independently execute clinical actions, (2) the degree of delegated decision authority, (3) the required form of human oversight, (4) the scope and constraints of the COD, and (5) the reversibility and potential clinical impact of failure. Table 2 provides a preliminary operational checklist intended to support more standardized autonomy assignment across dental AI systems.

**Table 2:** Preliminary operational checklist for assigning autonomy levels to dental AI systems.

Criterion	L0–L1	L2	L3	L4	L5
Can the system independently execute a clinical action?	No	No	Yes, after clinician initiation	Yes, within bounded workflow	Yes, end-to-end
Is clinician approval required before execution?	Always	Always	Required before delegated execution	Required at checkpoints or exceptions	Not required in real time
Type of human oversight	Direct continuous control	Active review	Supervisory override capability	Checkpoint-based supervision	Post-hoc or institutional governance
Can the system modify the clinical environment?	No	No	Yes, within constrained COD	Yes, across multi-step workflows	Yes, autonomously
COD restriction level	Minimal or not applicable	Defined diagnostic/planning scope	Strict bounded COD	Narrow validated COD	Highly restricted COD
Failure reversibility	Highly reversible	Usually reversible	Partially reversible	Lower reversibility	Potentially irreversible
Primary accountability	Clinician	Clinician	Shared clinician-system responsibility	Shared institutional responsibility	Institutional/regulatory responsibility

AI, artificial intelligence; COD, Clinical Operating Domain.

Autonomy classification should be applied at the functional-task level rather than the overall device level. Hybrid systems may operate across multiple autonomy levels depending on the clinical function being evaluated.

This rubric is intended as a preliminary conceptual aid to improve consistency and reproducibility of autonomy assignment rather than as a formally validated scoring instrument. Future work should focus on empirical validation, inter-rater reliability assessment, and consensus refinement across clinical, technical, and regulatory stakeholders.

## 6 Discussion

The proposed L0–L5 autonomy framework presents a conceptual structure for interpreting the evolving role of AI in dental practice. Beyond classification, it highlights key implications for governance, clinical implementation, and evaluation pathways as autonomy increases. The following sections discuss these considerations in relation to responsibility, safety, and translational readiness.

### 6.1 Governance and human-centered implications

The progression from assistive AI to autonomous clinical systems represents not only a technological shift but a structural transformation of clinical governance, responsibility, and human–machine interaction. Within this context, the proposed L0–L5 framework provides a conceptual scaffold for moving beyond binary categorization of AI systems toward a graded, proportional governance model, in which oversight mechanisms scale with the degree of delegated decision authority. Rather than regulating all systems uniformly, governance can be aligned with functional autonomy, enabling more precise calibration of validation, monitoring, and accountability requirements (18–21).

A critical inflection point within this framework occurs at Level 3, where systems transition from advisory outputs to delegated execution. At lower levels (L1–L2), AI functions primarily as assistive or advisory clinical decision support, with clinicians retaining full interpretive authority. In some regulatory contexts, such systems may not qualify as medical devices if clinicians can independently evaluate their outputs (22, 23). However, once systems begin executing clinical actions within defined boundaries, they increasingly resemble Software as a Medical Device, thereby necessitating formal risk management, validation, and lifecycle governance (24, 25). This transition highlights the importance of autonomy as a regulatory determinant, rather than relying solely on intended use or technical description. Nevertheless, the precise regulatory threshold at which advisory systems transition toward Software as a Medical Device classifications may vary across jurisdictions and evolving regulatory frameworks.

The escalation of autonomy has direct implications for liability and accountability. At lower levels, responsibility remains largely clinician-centered. As autonomy increases, responsibility becomes progressively distributed across clinicians, developers, institutions, and regulatory bodies (26). At Levels 3 and above, execution errors may occur despite appropriate supervision, requiring shared accountability frameworks that extend beyond traditional malpractice models (18). At higher levels (L4–L5), clinicians may function primarily as supervisors rather than direct operators, further challenging existing legal and ethical paradigms.

From a human-centered perspective, increasing autonomy reshapes both clinician roles and patient experience. Systems operating at higher autonomy levels may generate patient-facing outputs such as triage recommendations, automated communication, or recall instructions that directly influence care pathways. These outputs should be considered safety-critical interfaces requiring governance, auditability, and version control. Informed consent processes must therefore evolve, ensuring that patients understand not only that AI is involved, but also the level of autonomy it exercises, particularly the distinction between advisory and executional systems. This further reinforces the need for human-centered AI design, where transparency, interpretability, and trust are treated as core system requirements rather than secondary features.

Equity considerations become increasingly important as autonomy scales. While higher-autonomy systems may expand access to care, particularly in underserved or resource-limited settings, insufficient validation across diverse populations risks amplifying existing biases and disparities. A human-centered framework must therefore prioritize inclusive validation, contextual adaptability, and equitable deployment to ensure that AI benefits are broadly distributed rather than reinforcing structural inequities in oral healthcare.

Finally, the fragmented digital infrastructure of dental practice introduces additional challenges. Heterogeneous electronic records, imaging systems, and laboratory platforms may act as both enablers and risk multipliers for autonomous systems (27). At higher autonomy levels, where AI systems can initiate tool-mediated actions across interconnected platforms, failures in interoperability, data consistency, or access control may propagate errors across clinical workflows. Accordingly, COD specification, auditability of system actions, and robust interoperability standards become essential safety requirements at Levels 3 and above (28).

### 6.2 Translational considerations for safe implementation

While defining autonomy levels provides conceptual clarity, safe integration of AI systems in dentistry requires a structured translational pathway aligned with increasing autonomy and risk. Systems should progress through staged validation and deployment phases that reflect both technical capability and governance maturity.

At early stages, assistive and advisory systems (L1–L2) should undergo rigorous analytical validation using retrospective datasets and expert reference standards. Beyond diagnostic accuracy, evaluation should incorporate human–AI interaction metrics, including decision latency, trust calibration, and susceptibility to automation bias. As systems approach conditional autonomy, transitional deployment strategies such as silent- or shadow-mode operation should be implemented. In these settings, AI outputs are generated in parallel with clinician decisions without influencing care, enabling assessment of real-world robustness, variability across clinical environments, and identification of edge cases prior to executional deployment.

For conditionally autonomous systems (L3), prospective clinical evaluation should be conducted, focusing on safety, interruption rates, supervisory workload, and system behavior under real-world conditions. As autonomy increases further (L4), evaluation should extend beyond safety to include clinical outcomes, complication rates, long-term effectiveness, and system reliability, alongside structured post-market surveillance and predefined update protocols for adaptive systems (19, 29, 30). Exploration of full operational autonomy (L5) should remain limited to highly constrained contexts, with strong ethical oversight and clearly defined governance boundaries.

Importantly, evaluation should extend beyond diagnostic performance alone. Validation should also account for specialty-specific and population-specific variability, including differences among pediatric, geriatric, medically complex, and healthy adult populations, as well as variability in imaging modalities, disease prevalence, cognitive status, systemic health, and procedural reversibility across dental disciplines (3, 5, 6). Increasing autonomy affects not only clinical accuracy but also workflow efficiency, resource allocation, clinician workload, patient experience, and access to care. In practical dental settings, AI-integrated workflows may include automated appointment triage, radiology reporting assistance, recall scheduling, laboratory communication, orthodontic monitoring, and CAD/CAM manufacturing coordination. The degree of autonomy may vary across workflow stages, requiring context-specific oversight, interoperability safeguards, and clinician verification checkpoints. A comprehensive assessment framework should therefore integrate clinical outcomes, operational performance, human factors, and ethical considerations, reflecting the broader impact of AI systems on care delivery.

### 6.3 Limitations and future directions

This Perspective presents a preliminary conceptual framework rather than an empirically validated model, and several limitations should be acknowledged. The proposed autonomy levels are normative and heuristic, informed by cross-domain taxonomies and regulatory principles but not yet validated through formal consensus methods or large-scale implementation studies. Importantly, the framework has not undergone prospective empirical validation and should currently be interpreted as a conceptual taxonomy rather than a clinically validated classification system. In practice, boundaries between levels may be fluid, particularly in hybrid systems spanning diagnostic, procedural, and workflow domains. Furthermore, the framework is function-based rather than system-based, recognizing that different components within a single AI system may operate at different autonomy levels depending on their role in perception, decision-making, or execution.

Autonomy is inherently context-dependent. A system classified at a given level in a low-risk workflow may require substantially stricter safeguards when applied to invasive procedures such as implantology or oral surgery. Accordingly, autonomy classification should be interpreted alongside task-specific risk stratification rather than as a fixed system attribute. Regulatory frameworks for AI in healthcare are also evolving. Ongoing developments in medical device regulation, adaptive algorithm governance, and liability structures may influence how autonomy is defined and operationalized over time. Furthermore, technical feasibility does not guarantee clinical acceptability. Adoption will depend on clinician trust, patient acceptance, and alignment with real-world clinical workflows.

Additionally, several technical limitations inherent to contemporary AI systems warrant further consideration as autonomy increases. Many dental AI models are trained on retrospective datasets derived from limited geographic regions, institutional settings, imaging devices, or patient demographics, thereby creating risks of dataset bias and uneven performance across diverse populations (31). Such biases may disproportionately affect underrepresented groups, including pediatric, geriatric, medically complex, or resource-limited populations, potentially reinforcing existing disparities in oral healthcare delivery (32). Generalizability also remains a major challenge. AI systems that demonstrate high performance under controlled development conditions may experience substantial performance degradation when deployed across different clinical environments, imaging protocols, electronic record infrastructures, or practitioner workflows. This is particularly relevant in dentistry, where heterogeneity in imaging quality, treatment approaches, and clinical documentation is common across institutions and countries. Accordingly, external multicenter validation and prospective real-world testing are essential before higher-autonomy deployment can be considered safe (33). Standardized benchmarking datasets and reporting frameworks may further improve reproducibility and comparability across autonomous dental AI systems.

Another important limitation relates to model calibration and uncertainty estimation. High predictive accuracy does not necessarily mean that AI confidence scores are well calibrated or clinically trustworthy. Poorly calibrated systems may generate overconfident outputs in unfamiliar or out-of-distribution scenarios, increasing the risk of automation complacency and delayed clinician intervention (34). As autonomy escalates, robust calibration methods, uncertainty-aware modeling, drift monitoring, and continuous post-deployment performance surveillance become increasingly important to ensure safe and reliable operation within defined CODs (19, 28, 29). Future research should focus on empirical validation of autonomy levels through consensus methodologies, simulation-based studies of human-AI interaction, and comparative evaluation of governance models. The framework should remain adaptive as digital dentistry evolves. Importantly, higher autonomy should not be assumed to be inherently preferable; in many clinical contexts, maintaining meaningful human control may remain both ethically and clinically optimal (20, 21).

## 7 Conclusion

Artificial intelligence in dentistry is progressing toward increasing levels of clinical autonomy, thereby necessitating clear conceptual, translational, and governance frameworks. The proposed L0–L5 autonomy taxonomy presents a conceptual approach for classifying dental AI systems according to agentic capability, delegated decision authority, human oversight, COD boundaries, and associated clinical risk. By identifying Level 3 as the critical transition point from advisory support to delegated execution, the framework highlights where regulatory, ethical, safety, and accountability requirements begin to intensify.

By linking autonomy levels to proportional governance and staged translational validation, this framework offers a preliminary conceptual foundation for the safe, transparent, and responsible integration of AI into oral healthcare. Future work should focus on empirical validation of the proposed taxonomy, refinement through multidisciplinary consensus, and alignment with evolving regulatory and ethical standards. In parallel, evaluating the real-world impact of increasing autonomy on clinical workflows, patient outcomes, clinician oversight, and healthcare equity will be essential. Ultimately, this framework may support future standardization efforts across research, regulation, clinical implementation, and responsible innovation in dental AI.

## Data Availability

Publicly available datasets were analyzed in this study. This data can be found here: Not applicable. No datasets were generated or analyzed in this study.
